# Associations between sexual activity and weight status: Findings from the English Longitudinal Study of Ageing

**DOI:** 10.1371/journal.pone.0221979

**Published:** 2019-09-09

**Authors:** Lee Smith, Lin Yang, Suzanna Forwood, Guillermo Lopez-Sanchez, Ai Koyanagi, Nicola Veronese, Pinar Soysal, Igor Grabovac, Sarah Jackson

**Affiliations:** 1 The Cambridge Centre for Sport and Exercise Sciences, Anglia Ruskin University, Cambridge, United Kingdom; 2 Deparmtent of Epidemiology, Center for Public Health, Medical University of Vienna, Vienna, Austria; 3 Department of Psychology, Anglia Ruskin University, Cambridge, United Kingdom; 4 Faculty of Sport Sciences, University of Murcia, Murcia, Spain; 5 Instituto de Salud Carlos III, Centro de Investigación Biomédica en Red de Salud Mental, CIBERSAM, Madrid, Spain; 6 National Research Council, Neuroscience Institute, Aging Branch, Padova, Italy; 7 Department of Geriatric Medicine, Faculty of Medicine, Bezmialem Vakif University, Istanbul, Turkey; 8 Department of Social and Preventive Medicine, Centre for Public Health, Medical University of Vienna, Vienna, Austria; 9 Department of Behavioural Science and Health, University College London, London, United Kingdom; Kasturba Medical College Mangalore, INDIA

## Abstract

**Objective:**

To investigate the association between weight status and sexual activity in middle-aged and older adults.

**Methods:**

Cross-sectional analysis on Wave 6 (2012/13) of the English Longitudinal Study of Ageing. Data were from 2,200 men and 2,737 women aged ≥50 years (mean 68.2 years). The explanatory variable was weight status, defined as normal-weight (BMI: ≤24.9), overweight (BMI: 25.0–29.9) or obese (BMI: ≥30) based on objective measurements of height and weight. Outcome variables were any self-reported sexual activity in the last year (yes/no) and, if yes, frequency of sexual intercourse in the last month. Covariates included a range of sociodemographic, lifestyle, and health-related variables. Associations were analysed using binary (past-year sexual activity) and ordinal (frequency of past-month sexual intercourse) logistic regression models.

**Results:**

The majority (73.3%) of men and half (50.0%) of women reported any sexual activity in the last year. The odds of reporting any sexual activity in the last year did not differ significantly by weight status in either men or women. However, among those who were sexually active, men with overweight (OR = 1.45, 95% CI 1.15–1.81, *p* = 0.002) or obesity (OR = 1.38, 95% CI 1.07–1.77, *p* = 0.015), and women with overweight (OR = 1.34, 95% CI 1.05–1.71, *p* = 0.017) reported significantly more frequent sexual intercourse in the last month compared with those who had a BMI in the normal-weight range, after adjustment for covariates.

**Conclusion:**

Older adults with overweight or obesity who are sexually active engage in more frequent sexual activity than those who are normal weight.

## Background

High prevalence of overweight and obesity is a leading public health concern in populations across the globe, with a substantial impact on physical and mental health.[1] For older adults in particular, carrying excess weight comes with additional health risks.[2] A substantial number of the medical complications associated with overweight and obesity become increasingly prevalent with age [3] and obesity exacerbates the age-related decline in physical function.[4] Maintaining regular participation in activities that involve a moderate-intensity level of physical activity can aid in the preservation of physical function in older age.[5]

A substantial body of work has demonstrated a negative association between body weight and physical activity in later life, with older adults with obesity tending to be less physically active than those with a body mass index (BMI) in the normal-weight range.[6,7] However, these studies have focused on conventional forms of physical activity, such as time spent walking, cycling, or engaging in housework. Another activity that meets the definition of moderate-intensity physical activity, but may be overlooked in existing studies of physical activity in relation to weight status, is sexual activity. In a study of young healthy couples, mean energy expenditure during sexual activity was 3.6kCal/minute and the mean intensity was 5.8 METS; leading the authors to conclude that sexual activity could be considered significant exercise.[8]

A growing literature has documented associations between engaging in sexual activity and better health and wellbeing. Studies have shown that a higher frequency of sexual activity is associated with a number of benefits for physical and mental health, including a reduction in cardiovascular events in later life, reduced risk of fatal coronary events, prostate and breast cancer,[9–11] and better reported quality of life.[12] While the cross-sectional design employed by the majority of these studies means it is not clear whether sexual activity promotes good mental and physical health or whether good mental and physical health promotes a higher frequency of sexual activity (or indeed, whether the relationship is bidirectional), there are plausible mechanisms by which sexual activity may be beneficial for health and wellbeing. First, as outlined above, sexual activity can be considered a form of physical activity [8] and thus those who engage in regular sexual activity likely yield the mental and physical health benefits acquired from a physically active lifestyle.[13] Second, during sexual activity or at the time sexual intercourse is at its peak, there is a release of endorphins, endogenous opioid peptides that function as neurotransmitters, which generates a happy or blissful feeling.[14] Circulating endorphin levels have been showing to be associated with higher natural killer cell activity.[15] A higher natural killer cell activity may be associated with a lower risk of cancer and viruses; they have also been found to prevent against infections of the lungs and play an important role in improving asthma and many other conditions.[16]

Studies indicate that there tends to be a decline in sexual activity with age. In a population-based study of English adults, sexual activity was found to decrease substantially from 50–59 years to ≥80 years in both men (94.1% to 31.1%) and women (53.7% to 14.2%).[17] A similar trend and magnitude of decline were also observed in a US population-based study.[18] While these studies were cross-sectional and cohort effects cannot be ruled out, an age-related decline in sexual activity is consistent with the wealth of longitudinal evidence documenting an age-related decline in physical function.[17,18] Preventing such a decline in older adults may aid in the preservation of health and wellbeing, but the extent to which sexual activity is modifiable is unclear and likely depends on the drivers of declines in sexual activity in later life. For example, the death of one’s partner is much more likely at older ages. Understanding the extent to which sexual activity varies as a function of weight status in this population group, after controlling for potential confounders, is important to gauge the extent to which it may be possible to improve the health and wellbeing of older adults with overweight and obesity by promoting continued engagement in sexual activity.

This study therefore aimed to examine the association between weight status and sexual activity in a population-based sample of older adults living in England. Specifically, we analysed differences between individuals who were normal-weight, overweight and obese in the prevalence of any sexual activity in the last year, and the frequency of sexual intercourse in the last month among those who were sexually active.

## Materials and methods

### Study population

Data were from the English Longitudinal Study of Ageing (ELSA), a population-representative longitudinal panel study of men and women aged ≥50 years living in England.[19] Participants take part in biennial assessments, in which they complete a computer assisted personal interview and self-completion questionnaires, with a nurse visit in alternate (even) waves to collect objective measures of health status, including height and weight. This study uses data from Wave 6 of ELSA (2012/13) as this was the only available wave at the time of analysis that included assessment of sexual activity (these measures have since been repeated in Wave 8). We restricted our sample to those who had complete data on sexual activity, BMI, and all covariates. All participants gave full informed consent to participate in the study, and ethical approval was obtained from the London Multi‐Centre Research Ethics Committee.

### Measures

#### Measurement of exposure

Weight was measured by nurses to the nearest 0.1 kg using portable electronic scales, and height was measured to the nearest millimetre using a portable stadiometer. Waist circumference was measured at the midpoint between the lower rib and the upper margin of the iliac crest using a tape with an insertion buckle at one end. The measurement was taken twice, using the same tape, and was recorded to the nearest even millimetre. Those whose waist measurements differed by more than 3 cm had a third measurement taken. The mean of the two valid measurements (the two out of the three measurements that were closest to each other, if there were three measurements) was used in the analyses. Nurses recorded any factors that might have compromised the reliability of the measurements (e.g., participant was stooped/unwilling to remove shoes) and these cases were excluded. BMI was calculated as weight in kg divided by the square of height in metres, and categorised as underweight (<18.5), normal-weight (18.5–24.9), overweight (25.0–29.9) and obese (≥30). Due to low numbers, we included underweight participants in the normal-weight category for analysis.

#### Measurement of outcomes

Past-year sexual activity and frequency of past-month sexual intercourse were assessed with two items from the Sexual Relationships and Activities Questionnaire (SRA-Q). All men and women were asked “*Have you had any sexual activity (sexual intercourse*, *masturbation*, *petting or fondling) in the past year*?” (yes/no). Those who reported being sexually active in the past year (i.e. answered “yes” to the previous question) were asked “*How many times have you had or attempted sexual intercourse (vaginal*, *anal or oral sex) in the past month*?”, with the following response options: not at all, once in the past month, 2–3 times in the past month, once a week, 2–3 times a week, once a day, more than once a day. Due to low numbers of participants endorsing the latter three response options, we combined those reporting having or attempting sexual intercourse once a week or more for analyses. The SRA-Q was administered as a self-completion questionnaire and returned in a sealed envelope. Participants were advised that all responses would be kept anonymous.

#### Measurement of covariates

We included in our analyses a range of covariates, selected *a priori*, hypothesised to be independently associated with both the exposure (weight status) and outcome (sexual activity) of interest.

Demographic information included age, sex, ethnicity (white vs. non-white) and partner status (married/cohabiting, separated/divorced, widowed, or single/never married). We used household non-pension wealth quintile (calculated across all ELSA Wave 6 participants) as an indicator of socio-economic status, as this measure has been shown to be particularly sensitive in this age group.[20]

Health-related variables included self-reported limiting long-standing illness, smoking status (smoker vs. non-smoker) and frequency of alcohol intake, categorised as never/rarely (never–once or twice a year), regularly (once every couple of months–twice a week), or frequently (3 days a week–almost every day).[8] Physical activity was assessed with three items that asked participants how often they took part in vigorous, moderate and low-intensity activities (more than once a week, once a week, 1–3 times a month, hardly ever/never),[21] and further categorised into three groups, as previously described: inactive (no moderate/vigorous activity on a weekly basis); moderate activity at least once a week; and vigorous activity at least once a week.[22] Depressive symptoms were assessed using the 8-item Centre of Epidemiological Studies Depression (CES-D) scale, a scale highly validated for use in older adults.[23]

### Statistical analysis

Analyses were performed using IBM SPSS Statistics v.25. Data were weighted to correct for sampling probabilities and for differential non-response and to calibrate back to the 2011 National Census population distributions for age and sex. The weights accounted for the differential probability of being included in Wave 6 of ELSA and for non-response to the SRA‐Q. Details can be found at http://doc.ukdataservice.ac.uk/doc/5050/mrdoc/pdf/5050_elsa_w6_technical_report_v1.pdf.

Associations between weight status and covariates were assessed using one-way analyses of variance (ANOVAs) for continuous variables and chi-square tests for categorical variables. We then used logistic regression to analyse independent associations between weight status and sexual activity. We constructed two models for each outcome; an age-adjusted model, and a multivariable-adjusted model that included age, partner status, ethnicity, wealth, limiting long-standing illness, smoking status, alcohol intake, physical activity, and depressive symptoms. We used a binary logistic regression model to analyse data on any sexual activity in the past year in the whole sample, and ordinal logistic regression to analyse the frequency of sexual intercourse in the past month among those who were sexually active. All model assumptions were met. All analyses were performed separately for men and women on the basis of previous research demonstrating substantial differences in sexual activity between older men and women [17] and greater body image concerns among women than men [24].

We conducted three sets of sensitivity analyses in which we repeated the multivariable models: 1) excluding underweight participants, in order to evaluate the extent to which including underweight participants in the normal-weight category influenced the results; 2) further categorising participants with a BMI in the obese range into those with class I (BMI 30–34.9) vs. class II/III (BMI ≥35) obesity, in order to evaluate the extent to which associations differ at the extreme end of the weight spectrum; and 3) using elevated waist circumference as an alternative measure of adiposity, defined according to the National Cholesterol Education Program Adult Treatment Panel III waist circumference cut offs (≥102 cm in men, ≥88 cm in women).

## Results

Of the 10,601 people who were interviewed in Wave 6 of ELSA, 7,079 (67% of those eligible) returned the SRA-Q and 7,731 (72.9%) participated in the nurse visit in which height and weight were measured. Data on past-year sexual activity and BMI were available for 5,379 participants, of whom 4,937 (91.8%) had complete data on covariates and formed the final sample for analysis. Compared with the group of Wave 6 participants who were excluded, the analysed sample was significantly older on average (68.0 vs. 64.3 years, *p*<0.001) and more likely to be married (68.4% vs. 66.4%, *p* = 0.001), white (98.0% vs. 94.4%, *p*<0.001), and in the upper two quintiles of wealth (47.3% vs. 37.3%, *p*<0.001). They were less likely to smoke (10.4% vs. 14.2%, *p*<0.001), more likely to report drinking alcohol frequently (36.4% vs. 34.1%, *p* = 0.001), and less likely to be inactive (20.1% vs. 28.9%, *p*<0.001). A greater proportion had overweight or obesity (70.9% vs. 66.1%, *p*<0.001) and fewer were sexually active (62.5% vs. 73.8%, *p*<0.001).

There were 2,200 men (mean [SD] age = 67.8 [8.4] years) and 2,737 women (68.5 [8.8] years) in the analysed sample. A quarter (24.3%) of men had a BMI in the normal-weight or underweight range, 47.3% in the overweight range and 28.4% in the obese range. The distribution across weight categories was more even in women, with 31.8% in the normal-weight or underweight range, 35.0% in the overweight range and 33.3% in the obese range.

Sample characteristics are presented in Table 1. Among men and women, increasing weight status was associated with younger age (*p* = 0.021 in men, *p* = 0.016 in women) and lower wealth (*p*<0.001). Men who were overweight or obese were more likely to be married or cohabiting (*p* = 0.003) than normal-weight men, but partner status was not significantly associated with weight status in women (*p* = 0.304). Men and women who were overweight or obese were less likely to smoke (*p* = 0.003 in men, *p* = 0.001 in women). Men and women with obesity also had a higher rate of limiting long-standing illness (*p*<0.001), were more likely to be inactive (*p*<0.001) and reported the highest mean number of depressive symptoms (*p* = 0.002 in men, *p*<0.001 in women). Women with obesity were more likely to report drinking alcohol never or rarely than those who were normal-weight or overweight (*p*<0.001) but alcohol intake was not related to weight status in men (*p* = 0.378). There was no significant association between ethnicity and weight status in either men (*p* = 0.107) or women (*p* = 0.117), although the sample was almost exclusively white.

**Table 1:** Characteristics of the male and female samples in relation to weight status.

	Men				Women			
	Normal-weight (*n* = 544)^1^	Overweight (*n* = 1043)	Obese (*n* = 613)	*p*	Normal-weight (*n* = 894)	Overweight (*n* = 956)	Obese (*n* = 887)	*p*
Age (mean [SD] years)	68.50 (8.64)	68.05 (8.07)	67.23 (7.79)	0.021	68.05 (8.83)	68.84 (8.61)	67.71 (8.09)	0.016
Partner status								
Married/cohabiting	71.1	77.1	76.7	0.003	57.4	62.1	62.8	0.304
Separated/divorced	8.4	9.5	11.3	-	14.4	13.1	13.3	-
Widowed	10.1	7.3	6.5	-	22.4	21.0	19.3	-
Single/never married	10.5	6.0	5.6	-	5.8	3.9	4.6	-
Ethnicity								
White	95.0	94.9	97.1	0.107	97.7	96.8	95.8	0.117
Non-white	5.0	5.1	2.9	-	2.3	3.2	4.2	-
Wealth quintile								
1 (poorest)	13.2	12.1	19.2	<0.001	15.8	14.6	23.4	<0.001
2	16.1	18.1	18.3	-	17.1	19.4	23.4	-
3	21.0	20.6	24.1	-	18.4	21.7	24.1	-
4	23.1	25.1	21.5	-	20.2	23.1	19.0	-
5 (richest)	26.6	24.1	16.9	-	28.5	21.2	10.1	-
Limiting long-standing illness								
No	66.3	70.6	58.8	<0.001	66.5	68.7	50.1	<0.001
Yes	33.7	29.4	41.2	-	33.5	31.3	49.9	-
Smoking status								
Non-smoker	84.5	89.8	90.7	0.003	85.2	89.4	91.4	0.001
Smoker	15.5	10.2	9.3	-	14.8	10.6	8.6	-
Alcohol intake^1^								
Never/rarely	15.9	15.9	15.4	0.378	28.6	26.1	37.3	<0.001
Regularly	39.2	39.6	44.3	-	38.7	47.2	44.4	-
Frequently	44.9	44.6	40.2	-	32.6	26.6	18.4	-
Physical activity								
Inactive	18.4	17.9	24.1	<0.001	24.1	20.3	37.1	<0.001
Moderately active at least once a week	42.8	44.2	50.3	-	43.7	53.4	46.0	-
Vigorously active at least once a week	38.8	37.9	25.7	-	32.2	26.3	16.9	-
Depressive symptoms (0–8) (mean [SD] years)	1.02 (1.66)	0.89 (1.53)	1.19 (1.81)	0.002	1.34 (1.78)	1.31 (1.81)	1.75 (2.12)	<0.001

1 Unweighted sample sizes.

Values are percentages unless otherwise stated.

All figures are weighted for sampling probabilities and differential non-response.

SD = standard deviation.

2 Never/rarely = never–once or twice a year; regularly = once every 2 months–twice a week; frequently = 3 days a week–almost every day.

The majority (73.3%) of men and half (50.0%) of women reported any sexual activity in the last year. Table 2 summarises the prevalence of sexual activity and frequency of sexual intercourse in relation to weight status and Table 3 presents age- and multivariable-adjusted models of these associations. After adjustment for age, partner status, ethnicity, wealth, limiting long-standing illness, smoking status, alcohol intake, physical activity and depressive symptoms, there was no significant difference in the odds of any sexual activity in the last year by weight status. However, among those who reported being sexually active, men with overweight (OR = 1.45, 95% CI 1.15–1.81, *p* = 0.002) or obesity (OR = 1.38, 95% CI 1.07–1.77, *p* = 0.015) reported a higher frequency of sexual intercourse in the last month, relative to men who were normal-weight. Similarly, women with overweight reported a higher frequency of sexual intercourse in the last month than those who were normal-weight (OR = 1.34, 95% CI 1.05–1.71, *p* = 0.017), but there was no significant difference in the frequency of sexual intercourse between women with obesity and women who were normal-weight (OR = 0.99, 95% CI 0.77–1.29, *p* = 0.959).

**Table 2:** Prevalence and frequency of sexual activity in relation to weight status in men and women.

	Men			Women		
	Normal-weight (*n* = 544)^1^	Overweight (*n* = 1043)	Obese (*n* = 613)	Normal-weight (*n* = 894)	Overweight (*n* = 956)	Obese (*n* = 887)
Any sexual activity in the last year						
No	28.7	25.6	26.9	48.6	49.4	51.9
Yes	71.3	74.4	73.1	51.4	50.6	48.1
Frequency of sexual activity in the last month^2^						
Not at all	45.0	36.4	38.9	38.0	28.5	36.5
Once in the past month	19.2	18.0	19.3	19.0	20.9	20.7
2–3 times in the past month	18.9	23.5	21.0	22.3	26.6	23.0
Once a week or more	16.8	22.1	20.8	20.7	24.0	19.8

1 Unweighted sample sizes.

2 Among participants who reported being sexually active.

Values are percentages.

All figures are weighted for sampling probabilities and differential non-response.

**Table 3:** Age- and multivariable-adjusted associations between weight status and sexual activity in men and women.

		Men (*n* = 2,200)^1^				Women (*n* = 2,737)			
		Age-adjusted OR [95% CI]	*p*	Fully adjusted^2^ OR [95% CI]	*p*	Age-adjusted OR [95% CI]	*p*	Fully adjusted^2^ OR [95% CI]	*p*
Any sexual activity in the last year									
	Normal-weight	1.00	-	1.00	-	1.00	-	1.00	-
	Overweight	1.12 [0.84–1.48]	0.440	1.09 [0.81–1.46]	0.588	1.05 [0.84–1.32]	0.665	0.97 [0.76–1.25]	0.826
	Obese	0.91 [0.67–1.25]	0.565	1.02 [0.73–1.42]	0.918	0.82 [0.65–1.02]	0.076	0.98 [0.76–1.27]	0.879
Frequency of sexual activity in the last month^3^									
	Normal-weight	1.00	-	1.00	-	1.00	-	1.00	-
	Overweight	1.54 [1.23–1.93]	<0.001	1.45 [1.15–1.81]	0.002	1.40 [1.11–1.77]	0.005	1.34 [1.05–1.71]	0.017
	Obese	1.35 [1.06–1.73]	0.017	1.38 [1.07–1.77]	0.015	0.98 [0.77–1.25]	0.877	0.99 [0.77–1.29]	0.959

OR = odds ratio; CI = confidence interval.

All figures are weighted for sampling probabilities and differential non-response.

1 Unweighted sample sizes.

2 Adjusted for age, partner status, ethnicity, wealth, limiting long-standing illness, smoking status, alcohol intake, physical activity and depressive symptoms.

3 Among participants who reported being sexually active. Odds ratios reflect the odds of participants with overweight/obesity reporting more frequent sexual activity in the last month relative to normal-weight participants.

We performed three sets of sensitivity analyses. The first showed that excluding underweight participants (95 men and 142 women) from the normal-weight group did not alter the pattern of results. There was no association between weight status and any sexual activity in the last year in either men or women (OR range 0.91–0.99, *p*>0.6). Among men, overweight (OR = 1.48, 95% CI 1.17–1.87, *p* = 0.001) and obesity (OR = 1.41, 95% CI 1.08–1.83, *p* = 0.011) were associated with higher frequency of sexual intercourse in the last month, relative to normal-weight. Among women, overweight was associated with higher frequency of sexual intercourse in the last month (OR = 1.34, 95% CI 1.04–1.71, *p* = 0.023) relative to normal-weight, but there was no significant difference between obesity and normal-weight (OR = 0.99, 95% CI 0.76–1.30, *p* = 0.963).

The second set of sensitivity analyses showed that further sub-categorisation of weight status did not produce notably different results. We grouped participants in the obese category into those with class I (*n* = 441 men, 466 women) and class II/III obesity (*n* = 151 men, 303 women). There was no significant association in either men or women between class I obesity (men: OR = 1.05, 95% CI 0.74–1.49, *p* = 0.797; women: OR = 1.00, 95% CI 0.75–1.33, *p* = 0.972) or class II/III obesity (men: OR = 0.93, 95% CI 0.56–1.54, *p* = 0.771; women: OR = 0.96, 95% CI 0.68–1.34, *p* = 0.793) and any sexual activity in the last year, relative to normal-weight. Among men, class I obesity was associated with significantly higher frequency of sexual intercourse in the last month (OR = 1.36, 95% CI 1.04–1.78, *p* = 0.026) relative to normal-weight, and although it was not statistically significant a similar association was observed in men with class II obesity (OR = 1.42, 95% CI 0.96–2.11, *p* = 0.080). Among women, there was no significant association between class I (OR = 1.01, 95% CI 0.76–1.35, *p* = 0.941) or class II/III obesity (OR = 0.96, 95% CI 0.68–1.37, *p* = 0.826) and frequency of sexual intercourse.

The third set of sensitivity analyses showed a similar pattern of results using elevated waist circumference as an alternative indicator of adiposity. Elevated waist circumference (men: *n* = 980; women: *n* = 1,414) was not associated with any sexual activity in the last year in men (OR = 1.02, 95% CI 0.80–1.30, *p* = 0.892) or women (OR = 0.98, 95% CI 0.79–1.21, *p* = 0.817). Men with a waist circumference ≥102cm reported a higher frequency of sexual intercourse in the last month than those with a healthy waist circumference measurement, although the difference did not reach statistical significance (OR = 1.20, 95% CI 0.998–1.45, *p* = 0.053). There was no association between high waist circumference (≥88cm) in women and past-month frequency of sexual intercourse (OR = 1.01, 95% CI 0.82–1.24, *p* = 0.944).

## Discussion

In this large sample of older adults in England, the odds of reporting any sexual activity in the last year did not differ significantly between normal-weight, overweight and obese BMI groups. However, among those who were sexually active, men with overweight or obesity and women with overweight reported a higher frequency of sexual intercourse in the last month compared with those who were normal-weight. Similar results were observed when using waist circumference as an indicator of overweight/obesity.

Previous research has shown that older adults with overweight and obesity tend to be less physically active than their normal-weight counterparts [6,7]. However, we did not observe the same pattern with regard to sexual activity: rather, we found that sexually active older adults who carried excess weight engaged in more frequent sexual activity. As such, the scope to mitigate the adverse health impacts of overweight and obesity in later life may be limited by these groups already having higher levels of sexual activity.

A plausible explanation for these observed associations is that people in stable cohabiting relationships tend to gain weight over time. For example, it was found in a longitudinal cohort of working adults (1,209 men and 1,319 women) that marriage was associated with a significant two-year weight gain.[25] Other studies have found similar findings.[26,27] Interestingly, studies have also shown that separation from a spouse is associated with significant weight loss.[25] In a longitudinal study of 169 newlywed couples [28], spouses completed measures of height, weight, marital satisfaction, stress, steps toward divorce, and several covariates biannually for four years. Own and partner satisfaction were positively associated with changes in weight, and this association was mediated by steps toward divorce. Spouses who were less satisfied than usual or had partners who were less satisfied than usual were more likely to consider divorce and thus less likely to gain weight. The study’s findings challenge the idea that quality relationships always benefit health, suggesting instead that spouses in satisfying relationships relax their efforts to maintain their weight because they are no longer motivated to attract a mate.[28] This finding may explain the observed association in the present study; if individuals who are overweight are more likely to be in a happy and satisfying relationship, those who are in a satisfying and happy relationship are potentially more likely to engage in frequent sexual activity.

It should be noted here that women with obesity did not report a higher frequency of sexual intercourse. One plausible explanation may be that women with obesity find it more difficult to become sexually aroused. In women, the first sign of sexual arousal is an increase in the blood flow to the vaginal wall.[29] Diminished pelvic blood flow secondary to aortoiliac or atherosclerotic disease leads to vaginal wall and clitoral smooth muscle fibrosis. This can ultimately result in symptoms of vaginal dryness and dyspareunia,[30]) which may result in a decrease in sexual desire. Importantly, aortoiliac or atherosclerotic disease is associated with higher levels of adiposity.[31] Another possible pathway for the observed association is that women with obesity may have concerns about their body image and lack confidence this may subsequently reduce the amount of sexual activity in which they engage.[32]

To our knowledge, this is the first study to investigate the relationship between frequency of sexual activities and weight status. The large sample and objective measurement of weight status are clear strengths of the study. However, findings from the present study must be interpreted in light of its limitations. Sexual information was self-reported, and people may not respond honestly to questions for fear of being judged. However, participants were informed that survey responses would remain anonymous. Moreover, there is currently little other option to measure the exposure variables investigated in the present study other than by self-report. The measure used in the present study has also shown good criterion validity with several health outcomes.[33–35]) Although the present analysis controlled for many of the measurable risk factors predicting morbidity in older adults, it may not have perfectly controlled for the actual onset of a possibly still undiagnosed serious illness or frailty which may have influenced sexual activity outcomes. There were some missing data on body weight, either because it was not possible to obtain a valid measurement or because participants did not consent to provide this information. It is possible that those who were more self-conscious about their body weight were less likely to consent to weighing; and if this is related to sexual activity associations between higher weight status and sexual activity may have been over or underestimated. The cross-sectional design means the results do not provide any insight into how sexual activity changes in relation to changes in weight, or vice versa. It should be noted that since the completion of the present analyses further recent data on sexual activity has become available in the ELSA dataset. Future studies may now wish to examine this association using longitudinal models. Finally, there was a substantial amount of missing data and a number of differences between the analysed sample of participants and those who were excluded, which may limit the generalisability of the results to the older adult population in general.

In conclusion, the present results indicate that while the odds of older adults being sexually active did not differ by weight status, there were differences in the frequency of sexual intercourse among those who were sexually active. Men with overweight and obesity and women with overweight reported a higher frequency of sexual intercourse in the last month than those who were normal weight.
